# Comparison of acute kidney injury and clinical prognosis of vancomycin monotherapy and combination therapy with beta-lactams in the intensive care unit

**DOI:** 10.1371/journal.pone.0217908

**Published:** 2019-06-05

**Authors:** Soyoung Kang, Jimin Park, Yun Mi Yu, Min Soo Park, Euna Han, Min Jung Chang

**Affiliations:** 1 Department of Pharmaceutical Medicine and Regulatory Science, Colleges of Medicine and Pharmacy, Yonsei University, Incheon, Republic of Korea; 2 Department of Pharmacy and Yonsei Institute of Pharmaceutical Sciences, College of Pharmacy, Yonsei University, Incheon, Republic of Korea; 3 Department of Clinical Pharmacology, Severance Hospital, Yonsei University College of Medicine, Seoul, Republic of Korea; 4 Department of Pediatrics, Severance Hospital, Yonsei University College of Medicine, Seoul, Republic of Korea; University of Sao Paulo Medical School, BRAZIL

## Abstract

Antibiotics induced acute kidney injury (AKI) risk in critically ill patients is not well known. This study aimed to evaluate the AKI development and clinical outcomes in critically ill adult patients treated with vancomycin (VAN) or combined with piperacillin-tazobactam (TZP) or meropenem (MEM). This was a retrospective study on critically ill adult patients who were given VAN, TZP or MEM and maintained for at least 48 h. The risk of AKI development and clinical outcomes were compared using the simple analysis and multivariate logistic regression. Three hundred forty patients were eligible. The incidence of any AKI was significantly higher in patients treated with VAN + TZP than those with VAN + MEM or VAN alone (52.7% vs. 27.7% vs. 25.7%; p < .0001). The adjusted odds of AKI increased 2.43-fold in VAN + TZP versus VAN, but not different in VAN + MEM versus VAN. However, AKI duration and recovery rate were not statistically different. In addition, all-cause death within 30 days after AKI onset was not significantly associated with antibiotic regimens. AKI incidence is higher in critically ill patients administered with VAN + TZP than those with VAN + MEM or VAN. However, no obvious evidence was found to prove that antibiotic-induced AKI leads to poor clinical outcomes.

## Introduction

Acute kidney injury (AKI) is defined as a sudden decrease in kidney function involving both structural damage and loss of function [1]. According to Kidney Disease Improving Global Outcomes (KDIGO), AKI is defined as an increase in serum creatinine (SCr) level by ≥0.3 mg/dL within 48 h or by ≥50% from baseline that is occurring within the first seven days [2]. AKI frequently occurred in 8–22.7% of hospitalised patients, and the incidence was approximately 19.0–63.3% in critically ill patients [3–4]. AKI development was associated with increased hospital stay, mortality, and cost [5]. A study reported that a SCr level of >0.5 mg/dL was associated with >6.5-fold increase in mortality, an increase in hospital length of stay of 3.5 days, and an increase in average hospital cost >$5,000; therefore, AKI development should be prevented and controlled [5].

Critically ill patients are commonly given broad-spectrum antibiotics to manage both gram-positive and gram-negative bacteria [6]. Vancomycin (VAN) is usually combined with a beta-lactam such as piperacillin-tazobactam (TZP) or meropenem (MEM) [6–7]. VAN-associated renal toxicity has been reported, and AKI risk may be higher in those who received combination therapy with antipseudomonal beta-lactam antibiotics, such as TZP and MEM and some studies supported them [8–13]. Luther et al. reported that AKI incidence is higher in patients receiving VAN + TZP than those receiving VAN + cefepime (FEP)/carbapenem in a systematic review and meta-analysis [9]. However, FEP and carbapenems were grouped together among critically ill patients, making individual comparisons difficult in this study. A comparative study conducted on 10,236 patients in general wards reported that VAN + TZP has significantly higher nephrotoxicity than VAN + MEM [12]. On the other hand, comparisons on VAN and antipseudomonal beta-lactam especially MEM-induced AKI development for use more than 48 hours were limited, in addition, previous researches have been mostly restricted to compare concomitant clinical prognosis such as recovery rate and mortality in intensive care unit (ICU) patients [14,15]. Therefore, comparison studies of individual antibiotics combined with VAN in patients admitted to ICU were restricted until now.

This study evaluated the difference in AKI development such as incidence, duration and recovery, and concomitant clinical prognosis according to the antibiotic regimens administered in patients with VAN monotherapy or combination of VAN + TZP or MEM.

## Materials and methods

### Ethics

The study protocol was approved by the Severance Hospital Institutional Review Board (approval number: 4-2018-0715). Informed consent from patients was waived because this retrospective study did not exceed the minimal risk.

### Study design and population

This retrospective cohort study included critically ill adult patients (age ≥18 years) who were admitted to any ICUs, including the surgical, medical, cancer, or coronary departments of Severance Hospital, a 3,362-bed tertiary academic hospital in Seoul, South Korea, between January 2015, and December 2017.

The study included all patients who were not administered VAN, TZP, FEP, or MEM within 72 h before the ICU admission; started and maintained the treatment for at least 48 h after ICU admission; and had a starting interval between VAN and beta-lactams of less than 48 h as per hospital protocol. The antibiotic doses were adjusted for the patient’s kidney function based on the product information. The study excluded patients with previous severe renal disease, such as end-stage renal dysfunction, chronic kidney disease stage IV, kidney transplantation (ICD-10 code: N18.5, I12.0, N18.4, Z94.0, T86.1), and acute kidney injury (N17) 48 h before ICU admission. Patients with a baseline glomerular filtration rate of 30 mL/min/1.73 m^2^ or more according to the CKD-EPI creatinine equation were included, and those without baseline SCr data were also excluded.

### Clinical outcomes

Main outcomes were the AKI development, onset time, duration of AKI. AKI was defined based on KDIGO criteria as increased SCr level of 0.3 mg/dL within 48 h or ≥1.5 times than the baseline values [2]. AKI stage 2 was defined as 2.0‒2.9 times higher than the baseline; stage 3, 3.0 times higher than the baseline or increased SCr level of ≥4.0 mg/dL. The development of any AKI was observed during maximum 15 days of therapy or within 72 hours after the completion of therapy. Recovery was defined as SCr level restoration of <1.5 times the baseline. AKI duration was defined as days from onset to recovery.

In addition, concomitant clinical outcomes including recovery rate, all-cause death, length of hospital stay, and the need of dialysis were evaluated within 30 days after the AKI onset.

### Data collection

Clinical data, including demographics, comorbidities from 30 days before to 7 days after antibiotic treatment, SCr concentrations, concurrent nephrotoxic drugs during antibiotic treatment, and information on cultured bacteria from 30 days before to 7 days after antibiotic treatment, were extracted from the electronic medical records using the Clinical Data Retrieval System of Severance Hospital. The SCr concentration recorded as <0.2 mg/dL were substituted to be 0.2 mg/dL. Acute Physiology and Chronic Health Evaluation II (APACHE II) scores were collected within 24 h of ICU admission. Concurrent nephrotoxic drugs were categorised as follows: IV contrast, calcineurin inhibitors (cyclosporine and tacrolimus), nonsteroidal anti-inflammatory drugs, angiotensin-converting enzyme inhibitors, angiotensin II receptor antagonists, vasopressors (dobutamine, dopamine, epinephrine, and norepinephrine), diuretics (acetazolamide, amiloride, furosemide, hydrochlorothiazide, and spironolactone), and alkylating agents (cyclophosphamide, cisplatin, and ifosfamide). Baseline SCr concentrations were collected on the first day of antibiotic administration. If the SCr data were not recorded at the start of antibiotic administration, data before and after 24 h of antibiotic administration were used. The length of hospital stay was calculated from the initiation of antibiotic treatment until hospital discharge or all-cause death.

### Statistical analysis

All analyses were conducted with SAS ver. 9.4 (SAS Institute, Cary, NC, USA). Figures were drawn using the GraphPad Prism version 6.07 for Windows (GraphPad Software, La Jolla, CA, USA). The number of patients in the VAN + FEP group is too small to use statistically; therefore, only the other three groups (VAN alone, VAN + TZP, and VAN + MEM) were statistically analysed.

Continuous data among the three groups were compared using ANOVA or the Kruskal–Wallis test. Discrete data were compared using the chi-squared or Fisher’s exact test. Kaplan–Meier analysis and log-rank test were performed to compare AKI developmental probability. A p-value of <0.05 was considered statistically significant, which was adjusted using the Bonferroni correction.

Multivariate logistic regression was performed to evaluate effectiveness of variables associated with AKI development. Effective variable candidates were selected based on the p-value of <0.1 using simple analysis between those with and without development, and candidates were selected as follows: APACHE II score, antibiotics treatment group, sepsis and pneumonia among comorbidities, cardiogenic and septic shock, contrast and diuretics, and epinephrine, norepinephrine, dobutamine, and dopamine vasopressor among the concurrent nephrotoxic drugs. Demographic data, such as age, sex, and weight, were also included. Suitable variables were chosen using the LOGISTIC procedure and the stepwise selection using the SAS software. The probability model was the AKI group, and the goodness-of-fit of the multivariate logistic model was evaluated using the Hosmer–Lemeshow test [16].

## Results

### Patient characteristics

Table 1 shows the patient characteristics among those treated with VAN, VAN + TZP, and VAN + MEM. Among the 970 screened patients, 340 were eligible consisting of 183 in VAN, 74 in VAN + TZP, 83 in VAN + MEM. The median duration of antibiotic therapy was 7.0 days (interquartile range [IQR] 8.0).

**Table 1:** Patient characteristics according to antibiotics group.

Variables	Vancomycin^V^	Vancomycin + Piperacillin/tazobactam^VP^	Vancomycin + Meropenem^VM^	p-value	Post hoc
Sample size, n (%)	183	74	83	-	
Males, n (%)	121 (66.1)	55 (74.3)	50 (60.2)	0.174	
Age, years, mean ± SD	59.1 ± 16.4	60.1 ± 17.6	58.9 ± 15.9	0.884	
Weight, kg, mean ± SD	63.5 ± 13.0	65.3 ± 12.9	59.7 ± 11.5	**0.015**	V, VP > VM
BMI^a^, kg/m^2^, mean ± SD	23.0 ± 4.5	23.6 ± 4.0	22.6 ± 4.1	0.334	
APACHE II score, mean ± SD	21.0 ± 8.5	24.1 ± 8.2	24.5 ± 9.0	**0.003**	VM, VP > V
Serum creatinine, mg/dL, median (IQR)	0.7 (0.4)	0.9 (0.4)	0.7 (0.5)	**0.005**	VP > V, VM
eGFR, mL/min/1.73 m^2^, median (IQR)	97.8 (36.3)	86.8 (31.3)	95.8 (40.7)	**0.017**	V > VP
Vancomycin daily dose					
g/day, mean ± SD	1.9 ± 0.4	1.7 ± 0.6	1.8 ± 0.5	0.088	
mg/kg/day, mean ± SD	30.6 ± 10.2	27.3 ± 10.8	30.7 ± 11.1	0.060	
Beta-lactam daily dose, g/day, mean ± SD	-	14.0 ± 3.3	2.6 ± 0.6	-	
Interval of co-administration ≤24 h, n (%)	-	68 (91.9)	75 (90.4)	0.737	
Duration of Therapy, day, median (IQR)	8.0 (9.0)	6.5 (5.0)	8.0 (9.0)	0.136	
Length of hospital stay after start of antibiotics, day, median (IQR)	18.0 (26.0)	20.5 (34.5)	32.0 (45.0)	**0.001**	VM > V, VP
Comorbidities, n (%)					
Hypertension	85 (46.5)	31 (41.9)	31 (37.4)	0.369	
Diabetes mellitus	45 (24.6)	22 (29.7)	14 (16.9)	0.158	
Heart failure	18 (9.8)	16 (21.6)	15 (18.1)	**0.028**	VP > V
Sepsis	20 (10.9)	11 (14.9)	22 (26.5)	**0.005**	VM > V, VP
Pneumonia	15 (8.2)	11 (14.9)	13 (15.7)	0.122	
Shock, n (%)					
Cardiogenic	12 (6.6)	17 (23.0)	3 (3.6)	**< .0001**	VP > V, VM
Septic	14 (7.7)	9 (12.2)	18 (21.7)	**0.005**	VM > V
Hypovolemic	1 (0.6)	1 (1.4)	3 (3.6)	0.125	
Concurrent nephrotoxic drugs, n (%)					
Contrast	31 (16.9)	28 (37.8))	10 (12.1))	**< .0001**	VP > V, VM
Calcineurin inhibitor	7 (3.8)	2 (2.7)	10 (12.1)	**0.020**	VM > V
NSAID^b^	79 (43.2)	37 (50.0)	37 (44.6)	0.606	
ACE inhibitor^c^	15 (8.2)	5 (6.8)	5 (6.0)	0.801	
ARB^d^	20 (10.9)	8 (10.8)	1 (1.2)	**0.023**	V, VP > VM
Vasopressor					
Epinephrine	21 (11.5)	20 (27.0)	15 (18.1)	**0.009**	VP > V, VM
Norepinephrine	73 (39.9)	56 (75.7)	56 (67.5)	**< .0001**	VP, VM > V
Dobutamine	10 (5.5)	6 (8.1)	7 (8.4)	0.586	
Dopamine	1 (0.6)	9 (12.2)	2 (2.4)	**< .0001**	VP > V, VM
Diuretics	125 (68.3)	58 (78.4)	65 (78.3)	0.116	
Alkylating agent	1 (0.6)	1 (1.4)	0 (0)	0.448	
Amphotericin	7 (3.8)	1 (1.4)	9 (10.8)	**0.019**	VM > V, VP
Aminoglycoside	3 (1.6)	1 (1.4)	10 (12.1)	0.001	
Rifampicin	4 (2.2)	2 (2.7)	0 (0)	0.417	
Positive cultures, n (%)					
*Pseudomonas*	16 (8.7)	7 (9.5)	11 (13.3)	0.517	
*Acinetobacter*	24 (13.1)	4 (5.4)	25 (30.1)	**< .0001**	VM > V, VP
*Klebsiella*	13 (7.1)	9 (12.2)	23 (27.7)	**< .0001**	VM > V, VP
*Stenotrophomonas*	7 (3.8)	3 (4.1)	6 (7.2)	0.455	
*Enterobacter*	9 (4.9)	4 (5.4)	7 (8.4)	0.528	
*Escherichia*	17 (9.3)	1 (1.4)	13 (15.7)	**0.008**	VM, V > VP

The bold values indicate statistically significant difference (p-value < 0.05).

^a^BMI = Body mass index

^b^NSAID = Non-steroidal anti-inflammatory drugs

^c^ACE inhibitor = Angiotensin-converting-enzyme inhibitor

^d^ARB = Angiotensin II receptor blocker

The data were presented in mean ± standard deviation (SD) when the one-way analysis of variance (ANOVA) test was used according to their distribution of continuous variables; otherwise, median (interquartile range, IQR) was presented in Kruskal–Wallis test. However, the chi-square test was performed, or Fisher’s expected test was performed if the expected frequency was <5 for categorical variables. A post hoc test was performed using the adjusted Bonferroni correction.

Table 1 shows the patient characteristics among those treated with VAN, VAN + TZP, and VAN + MEM. The duration of antibiotic therapy and mean VAN daily dose among the three groups did not show significant differences. The APACHE II score was significantly lower in patients treated with VAN, which means that patients with more severe disease tended to receive VAN + TZP or VAN + MEM combination therapy. In addition, the combination of VAN + MEM therapy had been used for more severe infections than VAN or VAN + TZP, with higher proportion of sepsis, septic shock, and positive cultures, especially *Acinetobacter*, *Klebsiella*, and *Escherichia*. In addition, patients treated with VAN + MEM tended to stay longer in the hospital than VAN or VAN + TZP. Vasopressor was used in 220 ICU (62.9%) patients, particularly norepinephrine in 209 (59.7%) and epinephrine in 75 (21.4%) among the total participants. The vasopressor treatment was lowest in VAN (VAN vs. VAN + TZP, 49.2% vs. 83.8%, p < .0001; VAN + TZP vs. VAN + MEM, 83.8% vs. 73.5%, p = 0.118).

### Comparison of AKI development and clinical outcomes

**Fig 1:**
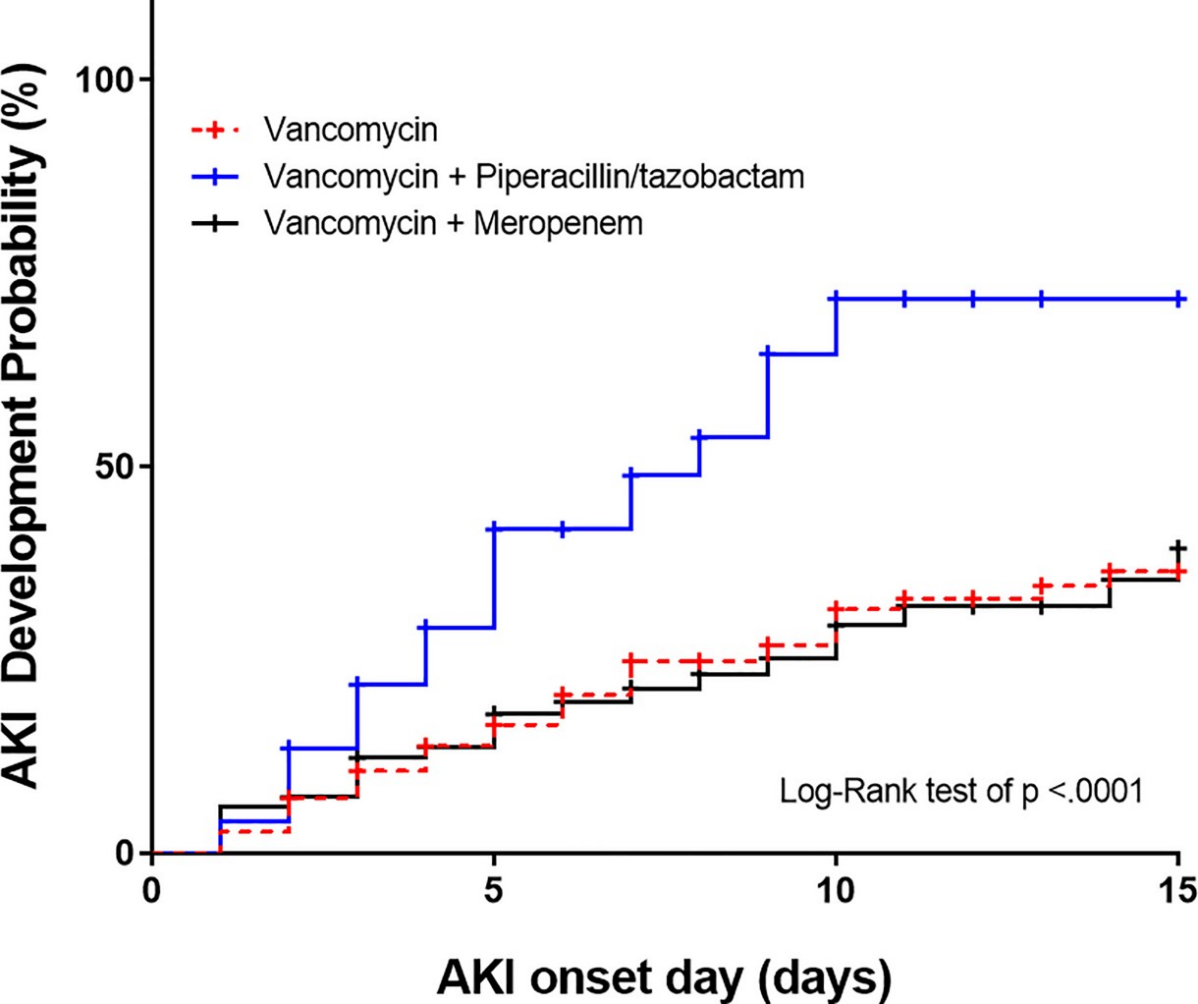
AKI development probability graph according to the survival analysis and log-rank test. Dotted red line marked indicates vancomycin monotherapy; continuous blue line indicates vancomycin plus piperacillin-tazobactam combination therapy; continuous black line represents vancomycin plus meropenem combination therapy; censored events were marked with crosses.

The AKI development defined by KDIGO in 114 patients (32.6%) consisted of 47 in VAN, 39 in VAN + TZP, 23 in VAN + MEM as presented in Table 2. In multiple comparisons to VAN, the AKI incidence was significantly higher in VAN + TZP (p < .0001) and similar in VAN + MEM (p = 0.728). The AKI stage 2 incidence was statistically higher in VAN + TZP than that in VAN + MEM or VAN alone (21.6% vs. 9.6% vs. 6.6%; p < .001). AKI stage 3 highly occurred in patients treated with VAN + TZP than those with VAN or VAN + MEM, but not statistically different (8.1% vs. 5.0% vs. 2.4%; p = 0.072). Kaplan–Meier analysis revealed a higher cumulative incidence of AKI in patients treated with VAN + TZP than those with VAN or VAN + MEM (Fig 1). The curve of AKI developmental probability was similar between VAN and VAN + MEM (p = 0.971), but not between VAN + TZP and VAN or VAN + MEM (p < .0001).

**Table 2:** Comparison between acute kidney injury development according to treatment group.

Outcomes	Vancomycin^V^	Vancomycin + Piperacillin/tazo-bactam^VP^	Vancomycin + Meropenem^VM^	p-value	Post hoc
Incidence of AKI, n (%)					
Any AKI	47 (25.7)	39 (52.7)	23 (27.7)	**< .0001**	VP > V, VM
AKI stage 1	26 (14.2)	17 (23.0)	13 (15.7)	**0.028**	VP > V
AKI stage 2	12 (6.6)	16 (21.6)	8 (9.6)	**< .001**	VP > V, VM
AKI stage 3	9 (5.0)	6 (8.1)	2 (2.4)	0.072	
Onset time to any AKI, days, median (IQR)	4.0 (5.0)	4.0 (5.0)	5.0 (7.0)	0.911	
Duration of AKI, days (IQR)					
Any AKI	2.0 (6.0)	3.0 (4.0)	2.0 (5.0)	0.821	
Stage 2 or 3	7.5 (11.5)	5.0 (6.0)	2.0 (8.0)	0.538	

The bold values indicate statistically significant difference (p-value < 0.05).

The median days of onset time to AKI were 4.0 (IQR 5.0) days in all patients, and the difference was not shown (VAN vs. VAN + TZP vs. VAN + MEM; 4.0 days vs. 4.0 days vs. 5.0 days; p = 0.911). The AKI onset persisted even after seven days and increased until approximately 10 days after initiating antibiotic therapy (Fig 1). The time of AKI incidence reaching to 50% was 8.0 days (95% CI 5.0‒9.0) in VAN + TZP.

A total of 78 patients (71.6%) recovered from AKI, and the highest recovery rate was achieved with VAN + TZP (84.6%), but it was not statistically significant (p = 0.071) (Table 3). The AKI duration in each treatment group was not statistically different in both any AKI (p = 0.821) and AKI stage 2 or 3 (p = 0.538). The median duration of any AKI was 2.0 days (IQR 5.0) in the three treatment groups, but AKI duration in stage 2 or 3 was slightly longer (median 6.0 days, IQR 8.0).

**Table 3:** Evaluation of clinical outcomes according to treatment group.

Outcomes	Vancomycin	Vancomycin + Piperacillin/tazobactam	Vancomycin + Meropenem	p-value
Recovery rate of AKI, n (%)				
Any AKI	31 (66.0)	33 (84.6)	14 (60.9)	0.071
AKI stage 2 or 3	12 (57.1)	17 (77.3)	7 (70.0)	0.563
Length of hospital stay after AKI onset, median (IQR)				
Any AKI	15.0 (26.0)	26.0 (48.5)	36.0 (81.0)	0.038*
AKI stage 2 or 3	33.0 (55.0)	21.5 (45.0)	92.0 (132.0)	0.206
Dialysis within 30 days after AKI onset				
Any AKI	3 (6.4)	2 (5.1)	2 (8.7)	0.886
AKI stage 2 or 3	1 (4.8)	1 (4.6)	1 (10.0)	0.793
All-cause death within 30 days after AKI onset, n (%)				
Any AKI	12 (25.5)	9 (23.1)	9 (39.1)	0.362
AKI stage 2 or 3	7 (33.3)	6 (27.3)	4 (40.0)	0.765

The bold values indicate statistically significant difference (p-value < 0.05).

*The result of Post hoc: Vancomycin + Meropenem > Vancomycin

Table 3 shows the evaluation of concomitant clinical outcomes according to the antibiotic treatment groups. All-cause death within 30 days after AKI onset was also similar among the three groups (25.5% in VAN, 23.1% in VAN + TZP, and 39.1% in VAN + MEM; p = 0.271). The length of hospital stay after AKI in patients treated with VAN + MEM was longer than those receiving other treatments (15.0 days in VAN, 26.0 days in VAN + TZP, 36.0 days in VAN + MEM; p = 0.038). Few patients required dialysis (n = 7, 6.4%), which was consistent among the three treatment groups (p = 0.886). In AKI stage 2 or 3, all outcomes were not different among treatment groups.

### Risk factor analysis

S1 Table shows a comparison of all variables, using simple analysis between the AKI and no-AKI group, to determine variables that affect the AKI development. Effective variables were the APACHE II scores, antibiotic treatment groups, sepsis status, and use of norepinephrine. The final model of the multivariate logistic regression is shown in Table 4. The area under the curve was 0.741, and the p-value of Hosmer–Lemeshow test was 0.659, which means this model fitted well. Risk factors that significantly affected the AKI development were sex, use of combination therapy of VAN plus TZP, sepsis, and use of norepinephrine (Table 4). The odds of AKI in VAN + TZP were significantly higher than VAN, but not between VAN + MEM and VAN. The APACHE II score was determined as an effective variable with p-value of less than 0.05, but was not as a powerful predictor of AKI. Norepinephrine use and sepsis increased the probability of AKI development. In addition, the risk of AKI development was higher in women than in men.

**Table 4:** Effective factors for development of AKI.

Variables	Univariate OR	95% CI		Multivariate OR	95% CI	
Treatment antibiotics						
Vancomycin + Piperacillin/tazobactam (ref. Vancomycin)	3.22	1.83	5.67	2.52	1.35	4.71
Vancomycin + Meropenem (ref. Vancomycin)	1.11	0.62	1.99	0.73	0.38	1.42
Demographics						
Female (ref. male)	1.47	0.91	2.36	2.13	1.21	3.72
Age	1.01	1.00	1.02	1.01	0.99	1.02
Weight	1.01	1.00	1.03	1.02	1.00	1.04
APACHE II score	1.06	1.03	1.09	1.04	1.01	1.08
With sepsis (ref. without sepsis)	2.37	1.30	4.30	2.32	1.19	4.52
Used norepinephrine (ref. unused norepinephrine)	3.31	2.01	5.44	2.03	1.15	3.59

OR = Odds ratio

CI = Confidence interval

The univariate OR was calculated according to the univariate logistic regression, and the multivariate OR was calculated according to the multivariate logistic regression.

## Discussion

In our study, the VAN + TZP had significantly higher AKI incidence than VAN or VAN + MEM (52.7% vs. 25.7% vs. 27.7%; p < .0001) in critically ill patients. In the multivariate logistic regression, including risk factor variables, the VAN + TZP group was shown to have higher odds than VAN or VAN + MEM. The median onset time and duration of AKI were also not different among all treatment groups. By contrast, concomitant outcomes within 30 days after AKI such as recovery rate, all-cause death, need of dialysis was also similar among all groups. The length of hospital stay was the longest in the VAN + MEM group after AKI, but this was shown also at the baseline. AKI was the most common in VAN + TZP, but it did not decisively influence the length of hospital stay.

This result of AKI incidence is fairly consistent with the previous reports on antibiotic nephrotoxicity. A large-scale retrospective study comparing VAN + TZP and VAN + MEM found that the former was associated with higher nephrotoxicity than the latter in non-critically ill patients [12]. In a sub-analysis among critically ill patients from a meta-analysis, Luther et al reported that VAN + TZP group showed higher nephrotoxicity than VAN monotherapy (OR = 9.62, 95% CI 4.48‒20.68), but not statistically different compared to VAN + FEP/carbapenem (OR = 1.43; 95% CI 0.83‒2.47) [9]. However, FEP and carbapenem plus VAN group was analysed as the same group and individual risk could not be shown and sample size of VAN + carbapenem was only 13 among 158 patients of VAN + FEP/carbapenem group. Moreover, they did not compare clinical outcomes among treatment groups followed by AKI. This is the first report that risk comparison of AKI and concomitant clinical outcomes by selection of TZP or MEM in critically ill patients.

We found that VAN + TZP significantly increased any AKI incidence including stage 2 or 3 after being administered for more than 48 h (median duration of treatment = 7.0 days). One recent paper reported that types of antipseudomonal beta-lactams used as a brief empiric therapy for less than 72 h with a median of 1.5 days did not affect the development of AKI stage 2 or 3 in ICU patients [15]. Although the duration of antibiotic treatment as empiric therapy is gradually decreasing, a multicentre ICU study reported that 50% of patients receiving empiric antibiotic therapy continued treatment for at least 72 h, and the probability of ventilator-associated pneumonia was lower with prolonged empiric antibiotic therapy (45.1% vs. 59.5%; p = 0.03) [6, 17]. In addition, the median duration of the unchanged empiric course was 5.5 days in an observational cohort study of empiric antibiotic therapy in six hospitals, and the rate of regimen changes within five days after initiating the empiric antibiotic therapy was reported to be less than one in three [18]. Therefore, this is a meaningful basis for an actual clinical situation by comparing the AKI incidence according to the antibiotic use for more than 48 h. To our knowledge, this is the first study that evaluated the AKI risks between VAN monotherapy and VAN plus MEM or TZP used more than 48 h in ICU patients.

The current study found that AKI incidence differs depending on antibiotic regimens, but did not have a decisive influence on poor clinical outcomes, such as the recovery rate and all-cause death and need of dialysis within 30 days after AKI onset (Table 3). This was also shown in the sub-analysis with AKI stage 2 or 3. In a previous study on non-ICU patients, AKI incidence was higher in the VAN + TZP group than the others, but did not directly result in poor clinical outcomes [11]. Although VAN’s nephrotoxic mechanism is presumably due to oxidative stress, that of TZP remains unclear [19, 20]. One study of critically ill patients reported that the renal filtration rate recovery was more delayed in TZP than MEM-treated patients while being exposed to the antibiotics (1.0 mL/min/1.73 m^2^/24 h vs. 2.9 mL/min/1.73 m^2^/24 h) [21]. But the renal filtration rate was recovered after discontinuing TZP (2.7 mL/min/1.73 m^2^/24 h), and they had shown that renal toxicity due to TZP was at least partially reversible [21]. We thought that renal function was partially restored by dose reduction or discontinuation of TZP, and then VAN + TZP-induced AKI did not primarily affect the clinical outcomes.

Critically ill patients are at high risk of developing AKI due to the complexity of concomitant nephrotoxic drugs, comorbidity, hemodynamic instability, or others [14, 22]. In this study risk factors that affect AKI development were female sex, sepsis, use of norepinephrine, and type of beta-lactam antibiotics (Table 4). This finding is consistent with previous studies, especially regarding sex and sepsis [23–27]. Women were estimated to have fewer glomeruli and nephrons than men, which may lead to a lower renal functional reserve [28]. Sepsis-induced AKI has been explained based on hypoperfusion, microvascular dysfunction, inflammation, and cellular response to inflammatory insults [27]. Contrary to that of the previous studies which suggested that most systemic hemodynamic parameters were not associated with AKI development, norepinephrine was one of risk factors in our study [29]. We suspect that hemodynamic status which needs norepinephrine use might have caused AKI in critically ill patients.

The percentage of sepsis in the VAN + MEM group was significantly higher than VAN or VAN + TZP (26.5% vs. 14.9% vs. 10.9%; p = 0.005), and the proportion of cultured bacteria with low susceptibility to antibiotics, such as *Acinetobacter*, *Klebsiella*, and *Escherichia* spp., was also high in VAN + MEM [30]. The length of hospital stay after antibiotic administration was also the longest in VAN + MEM, regardless of AKI incidence. According to the Sanford Guide to Antimicrobial Therapy 2018, the primary treatment option was MEM, not TZP, in systemic infection of *Acinetobacter baumannii*, *Klebsiella pneumonia* or *Escherichia* spp., and *Pseudomonas aeruginosa* [7]. Previous studies indicated severe infection frequently causes fatal conditions, and mortality was 27.4% even with adequate antibiotic administration [31, 32]. Thus, MEM tended to be used in severe infections compared to TZP, so treatment selection bias could be observed. However, even after controlling bias by logistic regression, VAN + TZP had more risk than VAN + MEM to cause AKI (Table 4). According to these data, we could infer that the choice of beta-lactam antibiotics as well as sepsis has a large impact on AKI regardless of other factors in ICU patients.

This study has some limitations. We defined the incidence of AKI based solely on the changed in SCr level because not all patients were measured the level of urine output, so the actual incidence of AKI may have been underestimated [33]. Although we tried to remove confounding factors and selection bias through multivariate logistic regression, the inherent limitation of a retrospective cohort research that various confounders could not be totally removed.

To our knowledge, this was the first study to assess the AKI incidence due to antibiotic regimens, such as VAN alone, VAN + TZP, and VAN + MEM in the ICU, which was associated with the comparison of clinical outcomes. This study provides new insights that the AKI occurred with VAN + TZP than those with VAN or VAN + MEM when antibiotics are used for longer than 48 h. This finding significantly indicated that antibiotics should be chosen in accordance with the patients’ clinical situation, rather than focusing on drug effect of AKI development in critically ill patients. In addition, kidney function recovery is higher in patients treated with VAN + TZP than those with VAN + MEM or VAN monotherapy in real clinical situations, which may be the basis of supporting the reversible nephrotoxicity of TZP.

## Conclusions

In conclusion, the AKI incidence in patients treated with VAN + TZP is higher than those treated with VAN monotherapy or VAN + MEM; however, this does not seem to have a decisive influence on clinical outcomes. AKI development and clinical outcomes of critically ill patients are presumed to be influenced not only by the antibiotic regimen, but also by infection severity, such as sepsis or sex. This study is expected to contribute to a deeper understanding of antibiotic nephrotoxicity and to provide meaningful results that will guide prescribing appropriate antibiotics to critically ill patients who might develop AKI.

## Supporting information

S1 Table Patient characteristics according to the development of acute kidney injury. ^a^BMI = Body mass index.
^b^NSAID = Non-steroidal anti-inflammatory drugs.
^c^ACE inhibitor = Angiotensin-converting-enzyme inhibitor.
^d^ARB = Angiotensin II receptor blocker.
The bold values indicate effective variable candidates associated with AKI development (p-value < 0.1).
* indicate screened variables to use multivariate logistic regression after stepwise selection.
The data were presented in mean ± standard deviation (SD) when the one-way analysis of variance (ANOVA) test was used according to their distribution of continuous variables; otherwise, median (interquartile range, IQR) was presented in Kruskal–Wallis test. However, the chi-square test was performed, or Fisher’s expected test was performed if the expected frequency was <5 for categorical variables.
(PDF)

S1 Data Minimal anonymized data set for statistical analysis. See “S1 data.csv”.
(CSV)

## References

[1] Makris K, Spanou L. Acute Kidney Injury: Definition, Pathophysiology and Clinical Phenotypes. Clin Biochem Rev 2016;37:85–98. 28303073 PMC5198510

[2] Kellum J a, Lameire N, Aspelin P, Barsoum RS, Burdmann E a, Goldstein SL, et al KDIGO Clinical Practice Guideline for Acute Kidney Injury. Kidney Int Suppl 2012;2:1–138. 10.1038/kisup.2012.7

[3] Sawhney S, Marks A, Fluck N, Levin A, Prescott G, Black C. Intermediate and Long-term Outcomes of Survivors of Acute Kidney Injury Episodes: A Large Population-Based Cohort Study. Am J Kidney Dis 2017;69:18–28. 10.1053/j.ajkd.2016.05.018 27555107 PMC5176133

[4] Wang HE, Muntner P, Chertow GM, Warnock DG. Acute kidney injury and mortality in hospitalized patients. Am J Nephrol 2012;35:349–55. 10.1159/000337487 22473149 PMC3362180

[5] Chertow GM, Burdick E, Honour M, Bonventre J V, Bates DW. Acute kidney injury, mortality, length of stay, and costs in hospitalized patients. J Am Soc Nephrol 2005;16:3365–70. 10.1681/ASN.2004090740 16177006

[6] Thomas Z, Bandali F, Sankaranarayanan J, Reardon T, Olsen KM, Critical Care Pharmacotherapy Trials Network. A Multicenter Evaluation of Prolonged Empiric Antibiotic Therapy in Adult ICUs in the United States. Crit Care Med 2015;43:2527–34. 10.1097/CCM.0000000000001294 26457751

[7] Gilbert DN, Chambers HF, Eliopoulos GM, Saag MS, Pavia AT. The Sanford guide to antimicrobial therapy 2018. 48th ed Sperryville, VA: Antimicrobial Therapy, Inc; 2018.

[8] Balcı C, Uzun Ö, Arıcı M, Hayran SA, Yüce D, Ünal S. Nephrotoxicity of piperacillin/tazobactam combined with vancomycin: should it be a concern? Int J Antimicrob Agents 2018;52:180–4. 10.1016/j.ijantimicag.2018.03.024 29649586

[9] Luther MK, Timbrook TT, Caffrey AR, Dosa D, Lodise TP, LaPlante KL. Vancomycin Plus Piperacillin-Tazobactam and Acute Kidney Injury in Adults: A Systematic Review and Meta-Analysis. Crit Care Med 2018;46:12–20. 10.1097/CCM.0000000000002769 29088001

[10] Navalkele B, Pogue JM, Karino S, Nishan B, Salim M, Solanki S, et al Risk of acute kidney injury in patients on concomitant vancomycin and piperacillin-tazobactam compared to those on vancomycin and cefepime. Clin Infect Dis 2017;64:116–23. 10.1093/cid/ciw709 27986669

[11] Robertson AD, Li C, Hammond DA, Dickey TA. Incidence of Acute Kidney Injury Among Patients Receiving the Combination of Vancomycin with Piperacillin-Tazobactam or Meropenem. Pharmacother J Hum Pharmacol Drug Ther 2018 10.1002/phar.2179 30175410

[12] Rutter WC, Burgess DS. Incidence of Acute Kidney Injury among Patients Treated with Piperacillin-Tazobactam or Meropenem in Combination with Vancomycin. Antimicrob Agents Chemother 2018;62:e00264–18. 10.1128/AAC.00264-18 29712661 PMC6021655

[13] AY M.S., Al Yami MS. Comparison of the incidence of acute kidney injury during treatment with vancomycin in combination with piperacillin–tazobactam or with meropenem. J Infect Public Health 2017;10:770–3. 10.1016/j.jiph.2016.11.007 28209320

[14] Hanrahan TP, Harlow G, Hutchinson J, Dulhunty JM, Lipman J, Whitehouse T, et al Vancomycin-Associated Nephrotoxicity in the Critically Ill. Crit Care Med 2014;42:2527–36. 10.1097/CCM.0000000000000514 25083977

[15] Schreier DJ, Pharm D, Kashani KB, Tootooni MS, Ph D, Rule AD. Incidence of acute kidney injury among critically ill patients with brief empiric use of anti- pseudomonal beta-lactams with vancomycin. Clin Infect Dis 2018. 10.1093/cid/ciy724 PMC7181379 30165426

[16] Archer KJ, Lemeshow S, Hosmer DW. Goodness-of-fit tests for logistic regression models when data are collected using a complex sampling design. Comput Stat Data Anal 2007;51:4450–64. 10.1016/j.csda.2006.07.006

[17] Baby N, Faust AC, Smith T, Sheperd LA, Knoll L, Goodman EL. Nasal Methicillin-Resistant Staphylococcus aureus (MRSA) PCR Testing Reduces the Duration of MRSA-Targeted Therapy in Patients with Suspected MRSA Pneumonia. Antimicrob Agents Chemother 2017;61 10.1128/AAC.02432-16 28137813 PMC5365699

[18] Braykov NP, Morgan DJ, Schweizer ML, Uslan DZ, Kelesidis T, Weisenberg SA, et al Assessment of empirical antibiotic therapy optimisation in six hospitals: an observational cohort study. Lancet Infect Dis 2014;14:1220–7. 10.1016/S1473-3099(14)70952-1 25455989 PMC5525058

[19] Elyasi S, Khalili H, Dashti-Khavidaki S, Mohammadpour A. Vancomycin-induced nephrotoxicity: mechanism, incidence, risk factors and special populations. A literature review. Eur J Clin Pharmacol 2012;68:1243–55. 10.1007/s00228-012-1259-9 22411630

[20] Bamgbola O. Review of vancomycin-induced renal toxicity: an update. Ther Adv Endocrinol Metab 2016;7:136–47. 10.1177/2042018816638223 27293542 PMC4892398

[21] Jensen J-US, Hein L, Lundgren B, Bestle MH, Mohr T, Andersen MH, et al Kidney failure related to broad-spectrum antibiotics in critically ill patients: secondary end point results from a 1200 patient randomised trial. BMJ Open 2012;2:e000635 10.1136/bmjopen-2011-000635 22411933 PMC3307126

[22] Spapen HD, Janssen van Doorn K, Diltoer M, Verbrugghe W, Jacobs R, Dobbeleir N, et al Retrospective evaluation of possible renal toxicity associated with continuous infusion of vancomycin in critically ill patients. Ann Intensive Care 2011;1:26 10.1186/2110-5820-1-26 21906376 PMC3224465

[23] Neugarten J, Golestaneh L, Kolhe N V. Sex differences in acute kidney injury requiring dialysis. BMC Nephrol 2018;19 10.1186/s12882-018-0937-y 29884141 PMC5994053

[24] Neugarten J, Sandilya S, Singh B, Golestaneh L. Sex and the risk of AKI following cardio-thoracic surgery: A meta-analysis. Clin J Am Soc Nephrol 2016;11:2113–22. 10.2215/CJN.03340316 27797892 PMC5142065

[25] Kanic V, Vollrath M, Kompara G, Suran D, Hojs R. Women and acute kidney injury in myocardial infarction. J Nephrol 2018;31:713–9. 10.1007/s40620-018-0504-4 29949012

[26] Bellomo R, Kellum JA, Ronco C, Wald R, Martensson J, Maiden M, et al Acute kidney injury in sepsis. Intensive Care Med 2017;43:816–28. 10.1007/s00134-017-4755-7 28364303

[27] Gómez H, Kellum JA. Sepsis-induced acute kidney injury. Curr Opin Crit Care 2016;22:546–53. 10.1097/MCC.0000000000000356 27661757 PMC5654474

[28] Luyckx V, Shukha K, Brenner BM. Inborn Nephron Diversity and Its Clinical Consequences. Rambam Maimonides Med J 2011;2:e0061 10.5041/RMMJ.10061 23908819 PMC3678805

[29] Legrand M, Dupuis C, Simon C, Gayat E, Mateo J, Lukaszewicz A-C, et al Association between systemic hemodynamics and septic acute kidney injury in critically ill patients: a retrospective observational study. Crit Care 2013;17:R278 10.1186/cc13133 24289206 PMC4056656

[30] Agodi A, Barchitta M, Quattrocchi A, Maugeri A, Aldisio E, Marchese AE, et al Antibiotic trends of Klebsiella pneumoniae and Acinetobacter baumannii resistance indicators in an intensive care unit of Southern Italy, 2008–2013. Antimicrob Resist Infect Control 2015;4 10.1186/s13756-015-0087-y 26539294 PMC4632366

[31] Kang C-I, Kim S-H, Park WB, Lee K-D, Kim H-B, Kim E-C, et al Bloodstream infections caused by antibiotic-resistant gram-negative bacilli: risk factors for mortality and impact of inappropriate initial antimicrobial therapy on outcome. Antimicrob Agents Chemother 2005;49:760–6. 10.1128/AAC.49.2.760-766.2005 15673761 PMC547233

[32] Angus DC, Linde-Zwirble WT, Lidicker J, Clermont G, Carcillo J, Pinsky MR. Epidemiology of severe sepsis in the United States: analysis of incidence, outcome, and associated costs of care. Crit Care Med 2001;29:1303–10. 11445675 10.1097/00003246-200107000-00002

[33] Wlodzimirow KA, Abu-Hanna A, Slabbekoorn M, Chamuleau RAFM, Schultz MJ, Bouman CSC. A comparison of RIFLE with and without urine output criteria for acute kidney injury in critically ill patients. Crit Care. 2012;16 10.1186/cc11808 23078781 PMC3682302

