# Supplementary material for: Comparison of acute kidney injury and clinical prognosis of vancomycin monotherapy and combination therapy with beta-lactams in the intensive care unit
Source: PLoS One. 2019 Jun 5;14(6):e0217908. doi: 10.1371/journal.pone.0217908 (PMC6550403; doi:10.1371/journal.pone.0217908)
Supplement: S1 Table — aBMI = Body mass index. bNSAID = Non-steroidal anti-inflammatory drugs. cACE inhibitor = Angiotensin-converting-enzyme inhibitor. dARB = Angiotensin II receptor blocker. The bold values indicate effective variable candidates associated with AKI development (p-value < 0.1). * indicate screened variables to use multivariate logistic regression after stepwise selection. The data were presented in mean ± standard deviation (SD) when the one-way analysis of variance (ANOVA) test was used according to their distribution of continuous variables; otherwise, median (interquartile range, IQR) was presented in Kruskal–Wallis test. However, the chi-square test was performed, or Fisher’s expected test was performed if the expected frequency was <5 for categorical variables. (PDF) [file pone.0217908.s001.pdf]

S1 Table. Patient characteristics according to the development of acute kidney injury

Characteristic	no-AKI	AKI	p-value
Sample size, n (%)	231 (67.9)	109 (32.1)	-
Male, n (%)	160 (69.3)	66 (60.6)	0.112*
Age, years, mean ± SD	58.4 ± 17.2	61.0 ± 14.8	0.184
Weight, kg, mean ± SD	62.3 ± 12.6	64.3 ± 13.1	0.166
BMI[a], kg/m^2, mean ± SD	22.9 ± 4.2	23.8 ± 4.4	0.098
APACHE II score, mean ± SD	21.2 ± 8.7	25.5 ± 8.0	**<.0001***
Serum creatinine, mg/dL, median (IQR)	0.75 (0.41)	0.74 (0.48)	0.742
eGFR, mL/min/1.73 m^2, median (IQR)	95.8 (34.7)	93.6 (34.5)	0.384
Vancomycin daily dose			
g/day, mean ±SD	1.8 ± 0.5	1.8 ± 0.5	0.929
mg/kg/day, mean ±SD	30.3 ± 10.6	29.1 ± 10.6	0.351
Piperacillin-tazobactam daily dose, g/day, mean ± SD	13.6 ± 3.6	14.4 ± 3.1	0.273
Meropenem daily dose, g/day, mean ± SD	2.7 ± 0.6	2.5 ± 0.7	0.296
Interval of co-administration ≤24 h, n (%)	226 (95.8)	110 (96.5)	0.745
Duration of therapy, day, median (IQR)	7.0 (9.0)	7.0 (7.0)	0.823
Length of hospital stay after start of antibiotics, day, median (IQR)	21.0 (29.0)	28.0 (39.0)	0.062
Comorbidities, n (%)			
Hypertension	96 (41.6)	51 (46.8)	0.429
Diabetes mellitus	54 (23.4)	27 (24.8)	0.885
Heart failure	29 (12.6)	20 (18.4)	0.210
Sepsis	27 (11.7)	26 (23.9)	**0.006***
Pneumonia	20 (8.7)	19 (17.4)	**0.029**
Shock, n (%)	35 (15.2)	35 (32.1)	**0.001**
Cardiogenic	10 (4.3)	22 (20.2)	**<.0001**
Septic	22 (9.5)	19 (17.4)	**0.056**
Hypovolemic	3 (1.3)	2 (1.8)	1.000
Concurrent nephrotoxic drugs, n (%)			
Contrast	38 (16.5)	31 (28.4)	**0.016**

Calcineurin inhibitor	14 (6.1)	5 (4.6)	0.765
NSAID[b]	97 (42.0)	56 (51.4)	0.132
ACE inhibitor[c]	17 (7.4)	8 (7.3)	1.000
ARB[d]	21 (9.1)	8 (7.3)	0.740
Vasopressor			
Epinephrine	26 (11.3)	30 (27.5)	**0.0003**
Norepinephrine	105 (45.5)	80 (73.4)	**<.0001***
Dobutamine	13 (5.6)	10 (9.2)	0.325
Dopamine	4 (1.7)	8 (7.3)	**0.022**
Diuretics	158 (68.4)	90 (82.6)	**0.009**
Alkylating agent	2 (0.9)	0 (0)	1.000
Amphotericin	12 (5.2)	5 (4.6)	1.000
Aminoglycoside	9 (3.9)	5 (4.6)	0.774
Rifampicin	3 (1.3)	3 (2.8)	0.390
Positive cultures, n (%)			
Pseudomonas	24 (10.2)	13 (11.4)	0.868
Acinetobacter	38 (16.1)	17 (14.9)	0.897
Klebsiella	24 (10.2)	24 (21.1)	**0.009**
Stenotrophomonas	10 (4.2)	6 (5.3)	0.875
Enterobacter	13 (5.5)	11 (9.7)	0.226
Escherichia	27 (11.4)	5 (4.4)	**0.051**

[a]BMI = Body mass index

[b]NSAID = Non-steroidal anti-inflammatory drugs

[c]ACE inhibitor = Angiotensin-converting-enzyme inhibitor

[d]ARB = Angiotensin II receptor blocker

The bold values indicate effective variable candidates associated with AKI development (p-value < 0.1).

* indicate screened variables to use multivariate logistic regression after stepwise selection.

The data were presented in mean ± standard deviation (SD) when the one-way analysis of variance (ANOVA) test was used according to their distribution of continuous variables; otherwise, median (interquartile range, IQR) was presented in Kruskal–Wallis test. However, the chi-square test was performed, or Fisher's expected test was performed if the expected frequency was <5 for categorical variables.
